# FOXP3 in Melanoma with Regression: Between Tumoral Expression and Regulatory T Cell Upregulation

**DOI:** 10.1155/2020/5416843

**Published:** 2020-10-23

**Authors:** Mirela Cioplea, Luciana Nichita, Daniela Georgescu, Liana Sticlaru, Alexandra Cioroianu, Roxana Nedelcu, Gabriela Turcu, Alin Rauta, Cristian Mogodici, Sabina Zurac, Cristiana Popp

**Affiliations:** ^1^Carol Davila University of Medicine and Pharmacy, Bucharest, Romania; ^2^Colentina University Hospital, Pathology Department, Bucharest, Romania; ^3^Colentina University Hospital, Hematology Department, Bucharest, Romania; ^4^Colentina University Hospital, Dermatology Department, Bucharest, Romania

## Abstract

Cutaneous melanoma is a significant immunogenic tumoral model, the most frequently described immune phenomenon being tumor regression, as a result of the interaction of tumoral antigens and stromal microenvironment. We present a retrospective cohort study including 52 cases of melanoma with regression. There were evaluated correlations of the most important prognostic factors (Breslow depth and mitotic index) with FOXP3 expression in tumor cells and with the presence of regulatory T cells and dendritic cells in the tumoral stroma. FOXP3 expression in tumor cells seems an independent factor of poor prognosis in melanoma, while regression areas are characterized by a high number of dendritic cells and a low number of regulatory T cells. FOXP3 is probably a useful therapeutical target in melanoma, since inhibition of FOXP3-positive tumor clones and of regulatory T cells could eliminate the ability of tumor cells to escape the immune defense of the host.

## 1. Introduction

Cutaneous melanoma (CM) is an aggressive skin tumor that can present a rare phenomenon in the absence of treatment: spontaneous regression, defined as complete or partial disappearance of malignant cells [1]. Although the mechanisms of regression in melanoma are not completely understood, current knowledge affirms that the host immune system has a key role in this process, histologically characterized by an intratumoral area where the malignant proliferation is, partially or completely, replaced by inflammatory cells, vascular hyperplasia, and fibrosis [2, 3]. Regression is described in 10-35% of melanomas, and it is difficult to be characterized in terms of prognosis significance; some studies suggest that it is a sinister event, probably linked to the enhancing effect that chronic inflammation has on tumorigenesis [4]. However, this immune process is the base of immunotherapy in melanoma with evident positive results [5, 6].

The etiology of regression is multifactorial and incompletely understood. Regression is an immune process mediated by CD8-positive cytotoxic T lymph cells, most likely triggered by an interaction between melanocyte-specific antigens and tumor-infiltrating lymphoid cells [7]. Practically, regression is the result of complex interactions between tumor cells and tumor microenvironment, the latter being composed of tumoral stroma, endothelial cell, leukocytes, fibroblasts, and extracellular matrix [8, 9]. Inflammatory cells in the tumor microenvironment have various phenotypes and functions (effector and suppressor T cells, B cells, natural killer cells, macrophages, and dendritic suppressor cells) and can express various immunological gene products that can modulate stromal microenvironment with significant impact on tumor local development [10, 11]. Studies show that in melanoma with favorable prognosis, intratumor inflammatory infiltrate is composed of numerous T cells CD3+, few B cells CD20+, few plasma cells CD138+, and variable Langerhans cells CD1a+ or langerin+ [12].

Due to the spontaneous regression, numerous tumor antigens, and antigen-induced specific antitumoral T cell responses, CM is a significant immunogenic tumor model [10]. Regulatory T cells (Treg) are an immunosuppressive subset of CD4+ T cells with an important role in decrease injurious immune-mediated inflammation in order to maintain self-tolerance. Treg express surface markers CD4 and CD25 and transcription factor forkhead box protein 3 (FOXP3) [10, 13]. In melanoma, a high number of regulatory T cells are present, usually in tumor microenvironment correlated with tumor immune escape [10, 14, 15]. Also, the ratio of CD8-positive T cells versus Treg in the tumor microenvironment has a predictive value for patients' survival [16, 17].

Dendritic cells (DC) are innate immune cells that process and present variable antigens to naïve T cells [18]. In tumors, DC have a suppressive role, but tumor microenvironment can block their antitumor actions, even inducing T cell tolerance and sustaining progressive tumor growth. The role of DC in CM is complex, DC representing a modulator of skin immunity, involved in both adequate immunological reactions and immune tolerance. Extremely important in antitumor immune defenses is the capacity of DC to present antigens to CD8+ T-lymphocytes via histocompatibility complex class I [19].

Thus, in melanoma, DC are especially found in areas of regression, being involved in the immune mechanisms that determine tumor cell destruction. Moreover, DC have significant patterns of distribution in areas of regression (nodular pattern) compared with areas without regression (predominantly diffuse pattern). These data suggest that DC are active players in melanoma's regression and could be used as therapeutic targets to enhance this natural process of tumor clearance [19].

Currently, the most important prognostic factors in CM are the Breslow depth of invasion and the proliferation index (mitosis count) [20, 21]; no immune parameters are being evaluated for this purpose to date.

FOXP3, a transcription factor, is mainly expressed in regulatory T cells and, also, in different tumors: melanoma, pancreatic carcinoma, and non-small-cell lung carcinoma [22–24]. In all these tumors, FOXP3 acts as a pathway to escape immune antitumoral response and represents a factor of increased aggressiveness, involved in tumorigenesis, progression, and metastasis [25, 26].

## 2. Materials and Methods

We present a retrospective cohort study including 52 consecutive cases of malignant melanoma of the skin with regression. The cohort included 30 men and 22 women, with age from 26 to 87 years (mean age about 46 years).

All cases were diagnosed on excisional surgical samples (only completely removed melanomas were included in our study), using routine histopathological techniques. Each lesion was largely sampled; then, tissue fragments were fixed using 10% buffered neutral formalin for 24 hours. After rinsing, a fully enclosed Leica tissue processor was used for paraffin embedding. From all blocks, there were obtained 2.5 *μ*m sections that were used for usual stain (hematoxylin-eosin) and for immunohistochemical tests. After the final diagnosis was formulated, each lesion was harvested in significant lesional areas (with and without regression) and included in multitissue blocks. From each multitissue block, there were performed sections for hematoxylin-eosin stain and immunohistochemical stains: FOXP3 and CD1a (Table 1).

The Breslow depth and mitotic index were evaluated during routine diagnosis on the entire lesion.

FOXP3 was evaluated in tumor cells using a semiquantitative scale (0: absent, 1: mild expression, 2: moderate expression, and 3: strong expression). Also, the distribution of positive tumor cells was evaluated.

In infiltrating lymph cells, the number of FOXP3-positive cells was evaluated, both in regressed and nonregressed areas as follows: rare (<20%), frequent (between 20% and 80%), and very frequent (>80%). Also, the distribution of FOXP3-positive lymph cells was evaluated in areas of regression and nonregressed areas.

Dendritic cells were evaluated on CD1a stain using a semiquantitative scale (0: absent, 1: rare, 2: frequent, and 3: very frequent), being described in regressed and nonregressed areas.

Obtained data were statistically processed using Microsoft Excel and Prism 8 software.

## 3. Results

### 3.1. Breslow Depth and Mitotic Index

**Figure 1:**
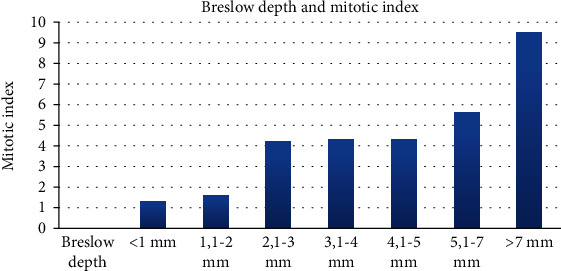
Distribution of the Breslow depth and mitotic index in our cohort.

In our group of melanomas with regression, the Breslow depth ranged between 0.2 mm and 14.3, mean value 2.75 mm. There were 18 thin melanomas (Breslow ≤ 1 mm) and 34 thick lesions (Breslow > 1 mm) (Figure 1).

**Figure 2:**
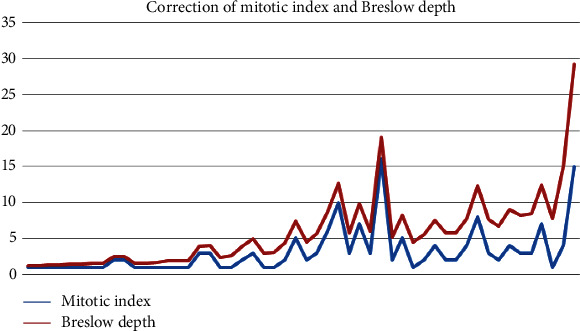
Correlation of the most important prognostic factor in melanoma.

The mitotic rate was between 1 and 16 mitotic figures on 1 mm^2^, with a mean mitotic index of 3.11 mitoses/mm^2^. As expected, there was a very strong correlation between the mitotic index and the Breslow depth (*t* test, two-tailed *P* value 0.0028) (Figure 2).

### 3.2. Expression of FOXP3 in Tumor Cells

**Figure 3:**
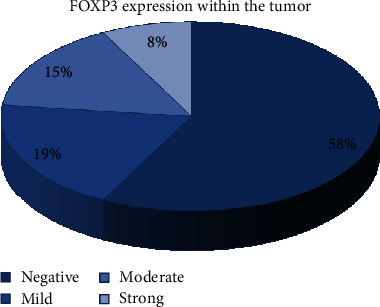
FOXP3 expression in tumor cells.

FOXP3 was expressed in tumor cells from 22 cases (10 mild expressions, 8 moderate expressions, and 4 strong expressions), while 30 cases were negative for FOXP3 (Figure 3).

**Figure 4:**
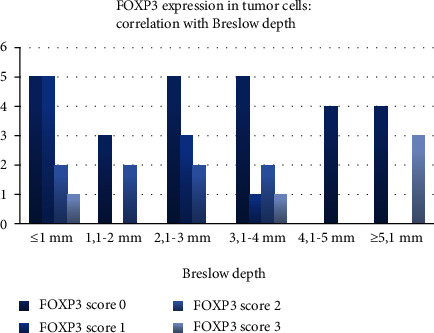
FOXP3 expression within the tumor—correlation with the Breslow depth.

FOXP3 expression in tumor cells was extremely statistically significant when correlated with the Breslow depth (*t* test, two-tailed *P* value < 0.0001). In thin tumors, FOXP3 was predominantly negative or had a mild expression, while in thicker tumors, there was a stronger expression of FOXP3 (Figure 4).

**Figure 5:**
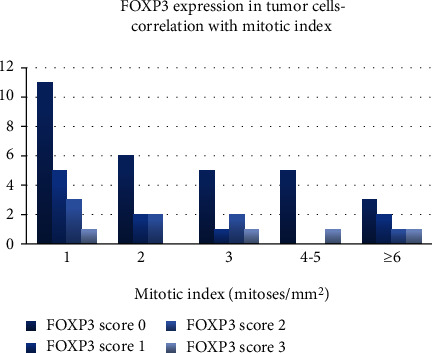
FOXP3 expression within the tumor—correlation with the mitotic index.

Also, the expression of FOXP3 in tumor cells was extremely statistically significant when correlated with a mitotic index (*t* test, two-tailed *P* value < 0.0001). Although most of the negative cases had a mitotic index of 2 or 3 mitoses/mm^2^, tumors with a high mitotic index showed an increased expression of FOXP3 in tumor cells than tumors with a low proliferation rate (Figure 5).

**Figure 6:**
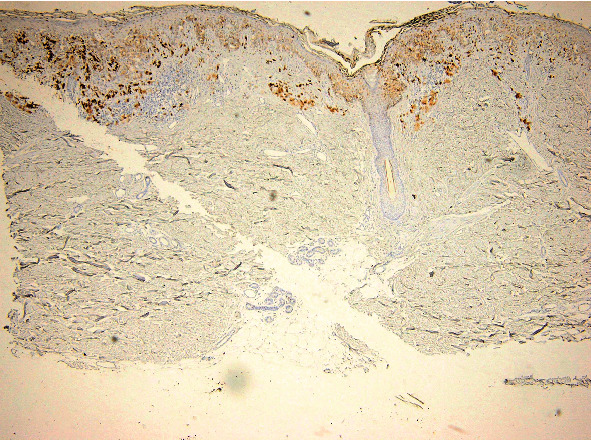
FOXP3 expression in melanoma: notice the presence of positive tumor cells and lymph cells. FOXP3 immunoassay, magnification 40x.

**Figure 7:**
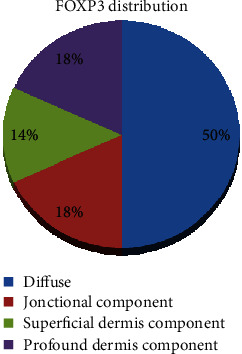
FOXP3 distribution within the tumor.

The distribution of positive tumoral cells (Figure 6) was evaluated in all 22 cases that showed positivity for FOXP3: 11 cases were diffusely positive, 3 cases were positive in tumoral cell confined in the superficial dermis, and 4 cases showed positivity in the junctional component, while 4 cases were positive in tumoral cell confined in the profound dermis (Figure 7).

**Figure 8:**
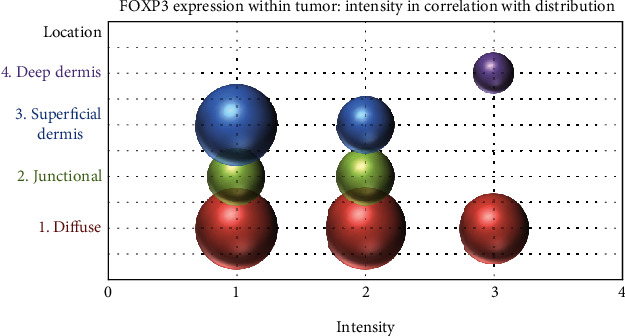
Correlation of FOXP3 intensity in tumor cells with the distribution of FOXP3-positive cells.

An interesting observation is that the intensity of FOXP3 expression was higher in lesions that showed a diffuse pattern of FOXP3 positivity, while tumors that had only focal positivity had a lower intensity of FOXP3 expression (without statistically significant correlation) (Figure 8).

### 3.3. Expression of FOXP3 in Intratumoral Lymph Cells

**Figure 9:**
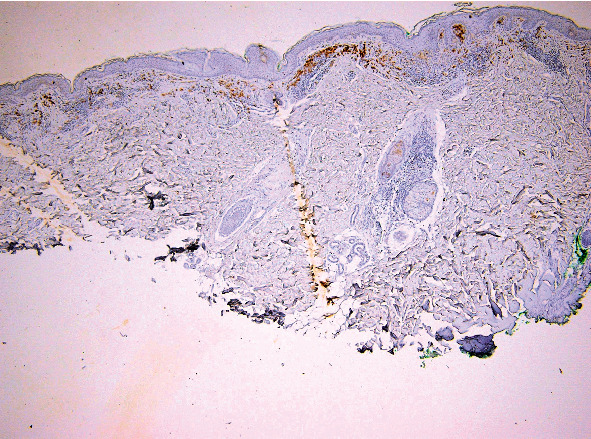
FOXP3 expression in lymph cells from an area of regressed melanoma. FOXP3 immunoassay, magnification 100x.

**Figure 10:**
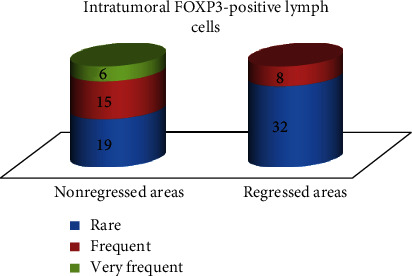
Distribution of FOXP3-positive lymph cells within the tumor.

Intratumoral lymph cell positive for FOXP3 (regulatory T cells) were identified in 40 cases, as follows: in areas without regression: rare in 19 cases, frequent in 15 cases, and very frequent in 6 cases; in regressed areas: rare in 32 cases and frequent in 8 cases (Figure 9). These data are extremely statistically significant when correlated (chi-square, two-tailed *P* value = 0.0001). In other words, we identified the lack of FOXP3-positive lymph cells in regressed areas (Figure 10).

Their presence is highly statistically significant when correlated with FOXP3 expression in tumor cells (*t* test, two-tailed *P* value = 0.0043)—tumors with a high expression of FOXP3 will have a high number of infiltrating FOXP3-positive lymph cells.

### 3.4. Dendritic Cells

**Figure 11:**
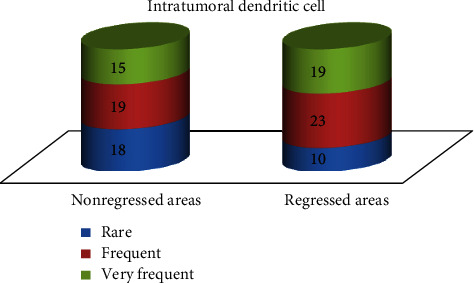
Distribution of dendritic cells within the tumor.

Dendritic cells were immunohistochemically stained with antibodies against CD1a and identified in all cases, as follows: in areas without regression: rare in 18 cases, frequent in 19 cases, and very frequent in 15 cases; in regressed areas: rare in 10 cases, frequent in 23 cases, and very frequent in 19 cases (Figure 11). The difference between regressed and nonregressed areas is statistically significant (chi-square, two-tailed *P* value = 0.0189).

**Figure 12:**
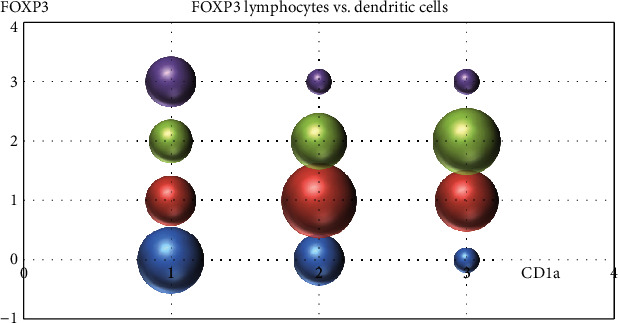
Correlation between the presence of intratumoral dendritic cells and FOXP3-positive lymph cells.

Their presence is extremely statistically significant correlated with the presence of intratumoral FOXP3-positive lymph cells (*t* test, two-tailed *P* value < 0.0001) (Figure 12).

## 4. Discussions

Correlation of the Breslow depth and mitotic index is well known in melanoma, both being markers of aggressive biological behavior. Practically, this correlation is so strong that recent studies sustain the idea that the mitotic index can be used as a surrogate to estimate Breslow thickness in incisional biopsies for planning surgical management [20]. These two features are mandatory for pathological reports in melanoma and are considered two of the most important prognostic factors in predicting the survival of melanoma patients [21]. Our study confirms this strong correlation and the importance of accurate evaluation of these parameters in diagnosis and research purposes. Thus, tumoral features that correlate with the Breslow depth and mitotic index are representing indicators of an aggressive behavior of the evaluated tumor.

The expression of FOXP3 in tumor cells is considered a mechanism of escaping immune destruction of malignant cells [25], being associated with tumorigenesis and tumor progression, being proposed as an independent prognosis marker in melanoma. The expression of FOXP3 is correlated with an early progression and a poor survival for patients with melanoma [27, 28]

In our study, the tumoral expression of FOXP3 was extremely statistically significant correlated with the Breslow depth, confirming its status as an important prognostic factor. These results confirm the hypothesis that FOXP3 tumor cells have a higher invasion potential and escape immune mechanisms. Our cohort included melanomas with regression, in which the immune response of the host actively destroys tumor cells. FOXP3-positive clones are resistant to this immune response and are responsible for thick, advanced tumors. This observation is supplementarily confirmed by the fact that FOXP3 overexpression is most frequently observed in tumors with a diffuse expression of FOXP3.

Also, FOXP3 expression in tumor cells correlated, in our study, with the mitotic index, sustaining the previously known fact that FOXP3 is implicated in tumor progression, mitotic actively tumors being mostly positive for FOXP3. Although some studies identified a suppressive effect of FOXP3 on melanoma cell lines (through growth inhibition and apoptosis activation) [29], our data are indicating that FOXP3 expression in tumor cells is correlated with a more aggressive biological behavior.

All cases had a significant number of intratumoral FOXP3 lymph cells, this being a mechanism through which melanoma is escaping the immune defense of the host. Multiple studies indicate that the high number of regulatory T cells positive for FOXP3 in melanoma is correlated with a poor outcome; these lymph cells are inhibiting the immune response of other T cells [10, 14]. In our study, FOXP3-positive lymph cells were upregulated only in areas without regression, while in areas with regression, they were rare. Subsequent studies are needed to demonstrate if FOXP3 regulatory T cells inhibit regression in melanoma, as part of the immunogenic effect of melanoma cells, or if the lack of FOXP3-positive cells is the result of regression.

The presence of intratumoral FOXP3-positive lymph cells was correlated, in our study, with FOXP3 expression in tumor cells. Probably, more aggressive FOXP3-positive clones of tumoral cells have a stronger ability to interfere with the immune antitumoral activity of T cells, one of the pathways inducing a T regulatory immunophenotype to intratumoral lymph cells. These data are important for the characterization of FOXP3 molecule as a therapy target [30], since not only positive tumor cells can be affected but also the immune defense against melanoma can be modulated via the regulatory T cell pathway.

Dendritic cells had an opposite behavior: they were more frequent in areas with regression. This observation was previously reported [19] and sustains the idea that dendritic cells are important players in antimelanoma immunity and tumoral regression. Also, this seems to be a peculiar pathway of immunity in melanoma, since in other tumors, there is a functional cross-talk between dendritic cells and FOXP3 regulatory T cells [31]. From this point of view, regression seems a process that is activated by dendritic cells and, at least partially, downregulated by regulatory T cells. Gai et al. identified a similar pattern in colorectal carcinoma, observing that an increased number of FOXP3-positive cells and a low number of dendritic cells correlate with tumor progression and ability to form lymph node metastasis [32].

## 5. Conclusions

FOXP3 is an interesting and promising molecular therapy target, since it seems to be an independent factor of aggressive behavior when it is expressed in tumor cells and, also, is deeply involved in modulating immune defense of the host.

Tumor regression is correlated with an inhibition of FOXP3 regulatory T cells in the presence of an increased number of dendritic cells. Data are suggesting that melanomas with a high number of regulatory T cells and a low number of dendritic cells have a higher risk for an aggressive biologic behavior.

## Figures and Tables

**Table 1:** Immunohistochemistry data.

Primary antibody	Clone	Host	Supplier	Dilution	Specificity
FOXP3	Monoclonal	Rabbit	ABCAM	1 : 50	Human
CD1a	MTB1 monoclonal	Mouse	Leica Biosystems	1 : 50	Human CD1a molecule

## Data Availability

The datasets used and/or analyzed during the current study are available from the corresponding author on reasonable request.
